# Reported snakebite mortality and state compensation payments in Madhya Pradesh, India, from 2020 to 2022

**DOI:** 10.1093/trstmh/trae045

**Published:** 2024-08-06

**Authors:** Priyanka Kadam, Bhupeshwari Patel, Maya Gopalakrishnan, Freston M Sirur, Omesh K Bharti, Amit Agrawal, Md Yunus, Dayal B Majumdar, Stuart Ainsworth

**Affiliations:** Snakebite Healing and Education Society, Mumbai 400050, India; Department of Trauma & Emergency Medicine, All India Institute of Medical Sciences, Bhopal 462026, Madhya Pradesh, India; Department of Medicine, All India Institute of Medical Sciences, Jodhpur 342005, Rajasthan, India; Department of Emergency Medicine, Kasturba Medical College, Manipal, Manipal Academy of Higher Education, Manipal 576104, Karnataka, India; Centre for Wilderness Medicine, Kasturba Medical College, Manipal, Manipal Academy of Higher Education, Manipal 576104, Karnataka, India; State Institute of Health & Family Welfare, Kasumpti, Shimla 171009, Himachal Pradesh, India; Department of Neurosurgery, All India Institute of Medical Sciences, Bhopal 462026, Madhya Pradesh, India; Department of Trauma & Emergency Medicine, All India Institute of Medical Sciences, Bhopal 462026, Madhya Pradesh, India; Calcutta National Medical College & Hospital, Kolkata 700014, West Bengal, India; Department of Infection Biology and Microbiomes, Institute of Infection, Veterinary and Ecological Sciences, University of Liverpool, Liverpool L3 5RF, UK

**Keywords:** compensation, India, Madhya Pradesh, snakebite, venom

## Abstract

**Background:**

India experiences the highest snakebite burden globally, with 58 000 predicted deaths annually. The central Indian state of Madhya Pradesh is thought to have a substantial snakebite burden and provides compensation to families who can demonstrate by postmortem and hospital treatment reports that their relatives have died due to snakebite. This study represents the first report on the frequency of distribution of compensation for snakebite deaths in Madhya Pradesh.

**Methods:**

Statewide snakebite death compensation data from 2020–2021 and 2021–2022, provided by the Madhya Pradesh health authorities, were analysed alongside interviews with 15 families that described the events that ultimately led to their compensation claims.

**Results:**

Compensation was paid to a total of 5728 families, with a total value equating to 22 912 Lakhs (approximately US${\$}$27.94 million). Families described commonly recognised snakebite risk factors and behaviours in the events that resulted in their relatives’ deaths.

**Conclusions:**

The snakebite burden in Madhya Pradesh is significant, both in terms of mortality and economic expenditure of the state. Sustained investment in preventative interventions, as well as monitoring of the rate of compensation payouts due to snakebite death as a measure of intervention effectiveness, should be considered to substantially reduce snakebite incidence and mortality.

## Introduction

Snakebite envenoming causes substantial global mortality and morbidity and is classified by the WHO as a neglected tropical disease.^1^ The burden of snakebite envenoming is thought to be the highest in India, which is estimated to experience approximately 58 000 deaths annually.^2^

Like snakebite envenoming in many other regions, the typical profile of Indian snakebite victims is one of rural poverty with limited access to healthcare facilities.^3–5^ In addition to the trauma experienced by victims and their families, the economic impact of snakebite can be severe for the immediate and wider family and, indeed, whole communities.^5,6^ Along with financial hardships, the psychological impact of snakebites on survivors and their families can be immense and long-lasting, leading to post-traumatic stress disorder.^7^

People experiencing snakebite tend to be of an age that is economically productive.^4^ The loss of workers, through death or chronic morbidity or through increased caring responsibilities, results in substantial economic losses for endemic countries. Interventions to limit snakebite envenoming, thereby reducing mortality and morbidity and improving the economic environment of a country, are predicted to be highly cost-effective if implemented.^8^ Therefore, there is a clear incentive for governments to take active steps in improving snakebite envenoming burdens.

Government compensation schemes for losses, both material and personal, caused by human–wildlife conflict, are common worldwide.^9,10^ India, with its mostly rural population and extremely diverse habitats and fauna, is no exception to this, with several human–wildlife conflict compensation schemes existing.^11^ Responsibility for implementing health policies and programmes lies with individual state governments, with several states providing compensation to families of those that have died of snakebite under provision of the National Disaster Management Act 2005.

To obtain compensation, families must submit claims to the local government where the incident occurred along with evidence of death resulting from a snakebite, most often a postmortem report. The application is then referred to the district's Disaster Authority Department. Once approved, compensation of a fixed amount, which varies state to state, is paid directly into the recipient's bank account.

One state that provides compensation in the event of a death due to snakebite is Madhya Pradesh. This large and highly populous central Indian state has high levels of poverty, with its population reliant on high levels of agricultural income.^12^ The Madhya Pradesh government pays Rs 4 Lakhs (approximately US${\$}$4880) for each successful snakebite death compensation claim. Considering that snakebite burden in the state is estimated to be high, with a predicted snakebite mortality rate of 6.7 per 100 000 of population,^2^ the economic burden of snakebite in terms of compensation payouts made by the state is expected to be substantial.

Here, we report figures of successful compensation claims paid by the Madhya Pradesh State Government as a result of snakebite deaths from 2020 to 2022. We also report the circumstances of 15 snakebites that resulted in successful applications for compensation after a death due to snakebite.

## Methods

### Data sources

Data on the number of successful snakebite death compensation payments and the total amount paid by health authorities in Madhya Pradesh in each district from 2020 to 2022 (2 y, spanning a period from April to March each year) were shared after a request to the Chief Secretary's Office, Madhya Pradesh State Government (Table 1). No data were excluded from the data supplied by the Madhya Pradesh State Government.

**Table 1:** Snakebite death compensation data by district supplied by the Madhya Pradesh health authorities

			
		2020–2021			2021–2022		
District	Population^a^	No. of deaths	Comp. snakebite death mortality rate/100 000 population	Comp. amount (Lakhs^b^)	No. of comp. deaths	Comp. snakebite death mortality rate/100 000 population	Comp. amount (Lakhs^b^)
Agar Malwa^c^	-	11	-	44	15	-	60
Burhanpur	892 720	11	1.2	44	10	1.1	40
Bhopal	2 845 228	12	0.4	48	15	0.5	60
Harda	644 168	17	2.6	68	13	2.0	52
Datia	850 409	18	2.1	72	14	1.6	56
Sheopur	737 721	19	2.6	76	39	5.3	156
Shajapur	1 713 211	20	1.2	80	13	0.8	52
Indore	3 822 846	22	0.6	88	21	0.5	84
Guna	1 501 492	27	1.8	108	21	1.4	84
Bhind	1 700 336	28	1.6	112	20	1.2	80
Neemuch	900 774	28	3.1	112	26	2.9	104
Sehore	1 459 484	32	2.2	128	38	2.6	152
Ujjain	2 239 868	33	1.5	132	22	1.0	88
Dewas	1 798 994	34	1.9	136	33	1.8	132
Hoshangabad	1 444 252	35	2.4	140	48	3.3	192
Gwalior	2 318 625	36	1.6	144	16	0.7	64
Rajgarh	1 670 967	36	2.2	144	46	2.8	184
Khandwa	1 547 611	37	2.4	148	50	3.2	200
Sidhi	1 404 980	37	2.6	148	38	2.7	152
Morena	2 087 311	38	1.8	152	49	2.3	196
Anuppur	781 836	39	5.0	156	27	3.5	108
Dindori	825 822	45	5.4	180	47	5.7	188
Narsinghpur	1 208 881	45	3.7	180	56	4.6	224
Ashok Nagar	825 771	55	6.7	220	37	4.5	148
Seoni	1 565 100	60	3.8	240	92	5.9	368
Balaghat	1 656 866	61	3.7	244	32	1.0	128
Katni	1 436 464	62	4.3	248	51	3.6	204
Singrauli	1 342 430	63	4.7	252	47	3.5	188
Tikamgarh	1 686 697	65	3.9	260	74	4.4	296
Ratlam	1 668 166	68	4.1	272	53	3.2	212
Shivpuri	1 873 073	68	3.6	272	88	4.7	352
Badwani	1 950 834	69	3.5	276	79	4.0	316
Umaria	709 996	69	9.7	276	60	8.5	240
Shahdol	1 258 597	70	5.6	280	62	4.9	248
Mandla	1 217 505	71	5.8	284	72	5.9	288
Vidisha	1 616 504	72	4.5	288	42	2.6	168
Khargone	2 124 182	75	3.5	300	78	3.7	312
Mandsaur	1 517 149	76	5.0	304	51	3.4	204
Raisen	1 473 165	80	5.4	320	64	4.3	256
Alirajpur	998 168	81	8.1	324	78	7.8	312
Dhar	2 493 541	81	3.2	324	63	2.5	252
Panna	1 182 818	85	7.2	340	72	6.1	288
Jabalpur	2 767 307	86	3.1	344	87	3.1	348
Rewa	2 679 829	88	3.3	352	79	2.9	316
Damoh	1 483 510	93	6.3	372	69	4.7	276
Betul	1 787 888	102	5.7	408	69	3.9	276
Chhindwara	2 384 690	105	4.4	420	111	4.7	444
Jhabua	1 458 753	115	7.9	460	92	6.3	368
Chhatarpur	2 142 329	129	6.0	516	109	5.1	436
Satna	2 523 898	146	5.8	584	160	6.3	640
Sagar	2 665 202	164	6.2	656	112	4.2	448
Total	82 887 968	3019	195	12 076	2760	177	11 040

a Population data are inferred from recent (October 2023) AADHAAR (a unique identifier number provided to citizens and resident foreign nationals, managed by the unique identification authority of India) figures, which are thought to cover the entire population of Madhya Pradesh.

b 1 Lakh=100 000 INR=approximately US${\$}$1219.5 (using an exchange rate of US${\$}$1= 82 INR).

c AADHAAR data for Agar Malwa are included in Shajapur district data.

Abbreviation: Comp., compensation.

### Data analysis

District heat maps of Madhya Pradesh were generated using Excel (Microsoft, Redmond, Washington, USA). The death rate or amount of compensation per 100 000 of population was determined by dividing the total number of deaths or total value of compensation in each district by the current predicted population for each district and multiplying by 100 000. Population data are inferred from recent AADHAAR (a unique identifier number provided to citizens and resident foreign nationals, managed by the unique identification authority of India) figures, which are thought to cover the entire population of Madhya Pradesh. AADHAAR data were accessed on 12 October 2023 from the Unique Identification Authority of India AADHAAR Dashboard (https://www.uidai.gov.in/aadhaar_dashboard/india.php?map_state=Madhya%20Pradesh).

### Exchange rate

For currency conversions, an exchange rate of US$1 to 82 Indian Rupees (INR) (the approximate conversion rate at the time of the study) was used.

### Compensation recipient interviews

Fifteen families who successfully applied for snakebite compensation were interviewed on their experiences. The interviews were performed in accordance with the ethical standards of the Helsinki Declaration of the World Medical Association. Ethical approval was granted by Sardar Patel University, Balaghat, Madhya Pradesh (SPU/PROJ/09/2022/055, dated 26 September 2022). After seeking informed consent from participants, a semistructured interview was conducted at those homes of families that received death-by-snakebite compensation. All the interviews were conducted by the study team and local trained volunteers in the local language, Hindi. The interviews lasted no longer than 30 min.

It is important to note that the interviews are not reflective of a bona fide qualitative investigation and are purely prospective in nature. Interviewee information was provided by state authorities for a limited number of individuals in a single district of Madhya Pradesh. No specific framework, sampling or coding was performed. The questions and topic guide used in all the interviews are available in the supplementary data. Each interview consisted of 28 questions, including details of the deceased, circumstances of the snakebite, health-seeking behaviour, the healthcare provided and its cost, the compensation-claiming process, how compensation was utilised and reflections on the experience. The interviews were recorded and answers transcribed before translation into English. A confidentiality agreement was signed by both of the local volunteers and the transcriber.

## Results

### Analysis of compensation due to snakebite death payments in Madhya Pradesh

**Figure 1.**
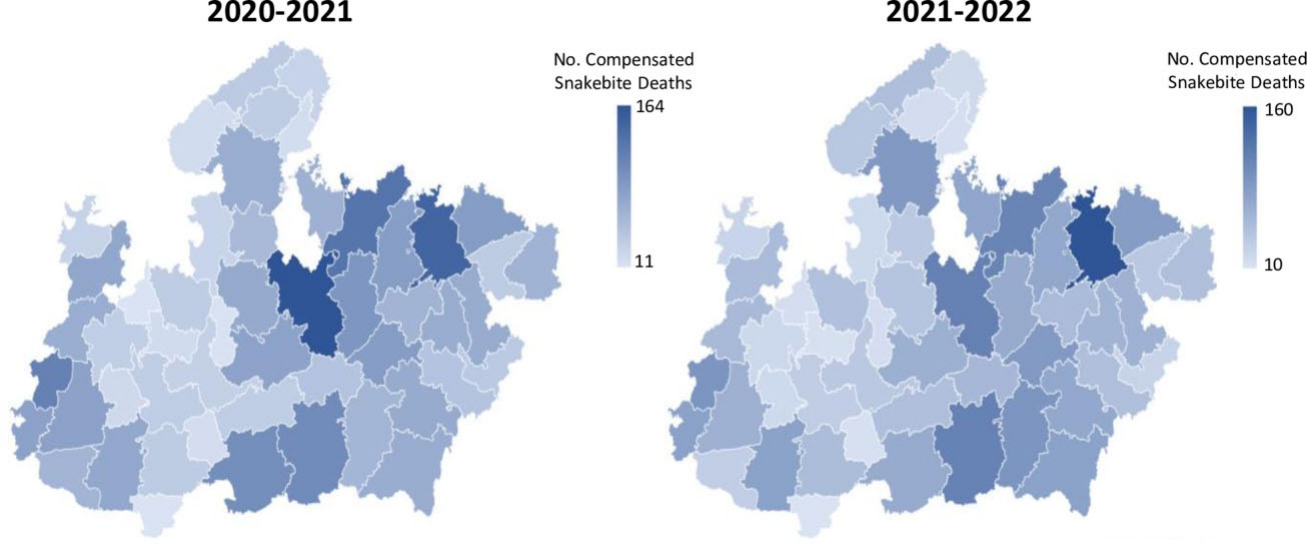
Microsoft Excel-generated heat maps of Madhya Pradesh depicting the total number of snakebite deaths compensated in each district in the 2-y period covered in this study (2020–2021, 2021–2022). The darker the blue colour, the greater number of compensated snakebite deaths.

Data supplied by the Madhya Pradesh health authorities revealed a total of 5779 snakebite deaths, resulting in compensation payments from 2020 to 2022 (April 2020 to March 2021: 3019; April 2021 to March 2022: 2760) (Table 1). A comparison of eligible snakebite deaths in the 2 y demonstrate a largely consistent number of deaths in each year examined (Table 1 and Figure 1). The districts with the highest number of snakebite death compensation payouts over the course of the study were Sagar (276) and Satna (306) (Table 1 and Figure 1). Total compensation paid by the Madhya Pradesh State Government over this 2-y period corresponds to 23 116 Lakhs, equivalent to US$28.2 million (using the approximate exchange rate at the time the study was conducted). Overall, the median specific compensated death mortality rate for Madhya Pradesh was 3.7 (range 0.4–9.7) deaths per 100 000 in 2020–2021 and 3.5 (0.5–8.5) deaths per 100 000 in 2021–2022.

### Experiences of the compensation recipients

**Figure 2.**
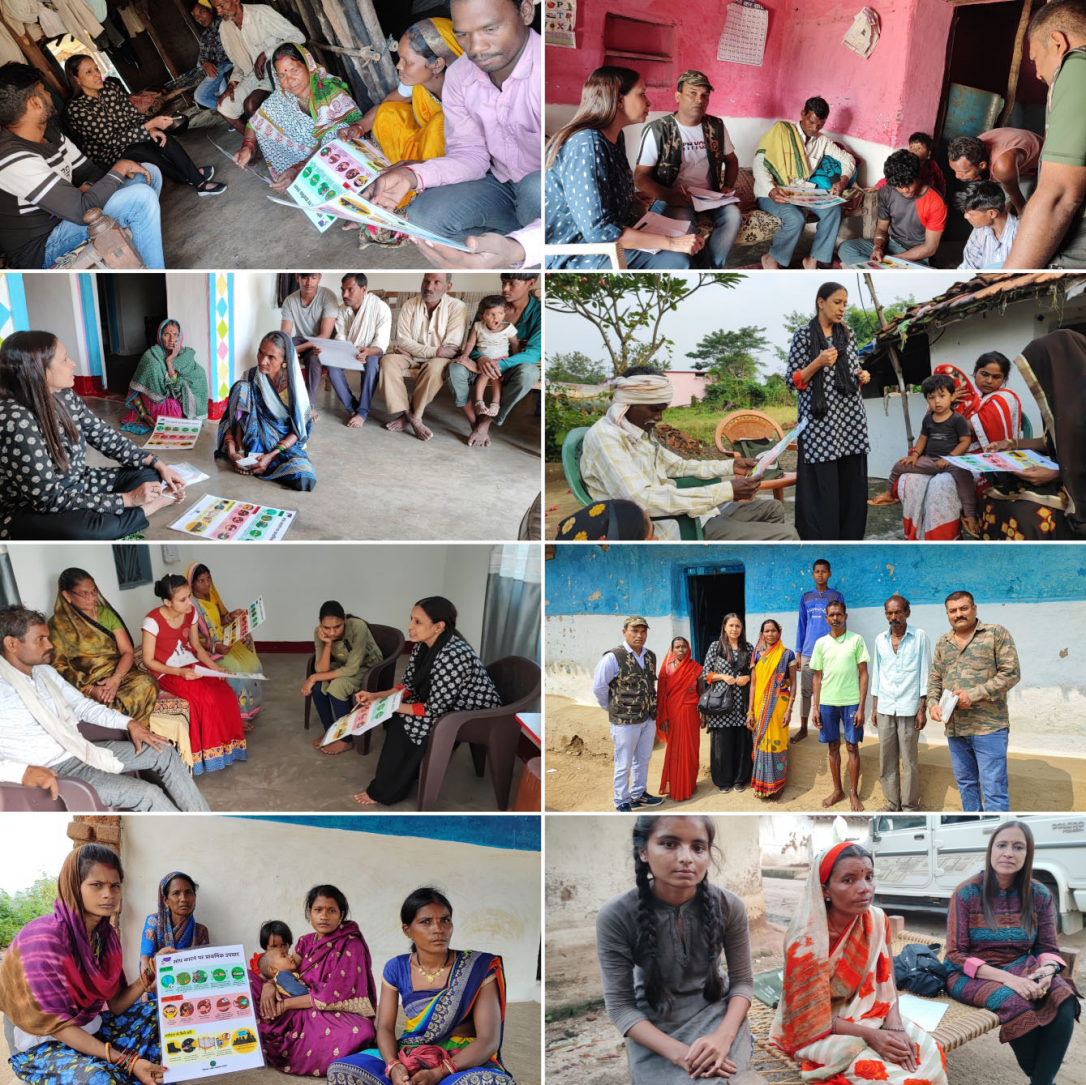
Photographic collage of the snakebite compensation recipients interviewed for this study. Written consent was provided by all participants.

Fifteen families that received compensation from the state government due to a snakebite death agreed to be interviewed to describe their experiences (Figure 2). Almost one-half of participants were illiterate. Descriptive data of the snakebite incident experienced by each family are provided in Table 2. The majority of snakebites occurred in the home (10/15), with most occurring while the victim was sleeping (7/15). The remainder (8/15) occurred while the victim was at work or preparing to cook food. All victims died within 24 h of the bite, with the approximate time to death ranging from 30 min to 20 h, with the majority (9/15) succumbing to the effects of envenoming within 4 h of the bite. Most snakebite victims (11/15) undertook some form of traditional healing prior to seeking healthcare. The majority of victims were transported to hospital in private vehicles, frequently motorbikes, or less so in ambulances. Delays in getting to hospital through waiting for transport were described by six participants. Seven patients were referred from the initial healthcare facility to a higher hospital, a journey which three victims did not survive.

**Table 2:** Details of snakebite incidence and compensation claims reported by 15 compensation recipients

Case	Gender	Age, y	Year	Time of bite	Time of death	Time to death	Bite setting	Faith healing	Referral to other HC	PM report issued^a^	Comp. applied^b^	Comp. received^c^
1	F	23	2021	18:00	19:45	1.5 h	Home	No	Yes	1 mo	n.d.	3–4 mo
2	F	12	2021	00:00	03:00	3 h	Home, asleep	No	Yes	15 d	15 d	2 mo
3	M	46	2021	13:00	13:30	0.5 h	Work	No	No	20 d	1 mo	3 mo
4	M	38	2021	14:00	17:00	3 h	Work	No	Yes	40 d	40 d	2 mo
5	F	30	2020	15:00	17:00	2 h	Home	Yes	No	1 mo	1 mo	5 mo
6	F	10	2021	23:00	00:30 (+1)	1.5 h	Home	Yes	Yes	n.d.	2 mo	5 mo
7	F	19	2020	22:00	05:00 (+1)	7 h	Home, asleep	Yes	No	15 d	15 d	2 mo
8	F	55	2020	02:00	08:00	6 h	Home, asleep	Yes	Yes	3 d	6 mo	2 mo
9	F	45	2020	01:30	03:30	2 h	Home, asleep	Yes	No	2 d	9 d	2 mo
10	F	21	2021	02:00	05:45	3.45 h	Home, asleep	Yes	No	8 d	15 d	1 mo
11	F	17	2021	04:30	06:00	1.5 h	Home, asleep	Yes	No	1 d	2 d	4 mo
12	F	23	2021	02:30	13:00	10.5 h	Home, asleep	Yes	No	15 d	n.d.	7 mo
13	F	22	2020	14:00	10:00 (+1)	20 h	Work	Yes	No	n.d.	n.d.	n.d.
14	M	48	2020	15:00	23:00	7 h	Work	Yes	Yes	>14 d	>2 mo	>1 y
15	M	47	2020	10:00	11:00	1 h	Work	Yes	Yes	20 d	2 mo	2 mo

Abbreviations: F, female; HC, health centre; M, male, n.d., not disclosed; PM, postmortem.

a Time after death for PM report to be obtained.

b Time after death for compensation claim submitted.

c Time to receive compensation after submission of claim.

Families reported waiting 4–40 d from the death of the snakebite victim until postmortem reports were available (Table 2). Compensation claims were typically paid after 30–60 d, except for to one family, who reported a wait of >1 y. Families described using the compensation for house building, funeral expenses for the deceased snakebite victim and household expenses.

## Discussion

This is the first study to analyse the extent of compensation claims paid to families of victims who died by snakebite in the Indian state of Madhya Pradesh. Over a period of 2 y (2020–2022), 5579 postmortem-verified snakebite deaths (an average of 2846 per year) were compensated by the Madhya Pradesh State Government (Table 1).

The figure of 2846 snakebite deaths per year, while substantial, is approximately 45% less than the recently predicted Madhya Pradesh snakebite mortality incidence of about 5200 deaths per year.^2^ When considering this discrepancy, it is important to consider that successful snakebite compensation claims will not be evidence of all deaths related to snakebites in the state, as they will not include families whose claims were unsuccessful or where claims were not submitted. Therefore, the compensation data presented here are almost certainly an under-representation of snakebite mortality in Madhya Pradesh. Conversely, the figure of 2846 compensated snakebite deaths/year is approximately nine times greater than the average of 330 snakebite deaths per year in Madhya Pradesh outlined official Government statistics within the National Health Profile.^13^ This stark disparity, due in part to the National Health Profile only reporting on hospital snakebite deaths,^13,14^ needs serious attention to enable a more accurate indication of snakebite mortality within the state.

The financial cost of compensation paid by the state of Madhya Pradesh is a considerable sum (Table 1), equating to a state expenditure, on average, of 1.3 billion rupees/year (about US$14.1 million). Previous estimates suggest that the rate of snakebite deaths will continue to remain substantial,^2^ thus a significant level of compensatory payouts is likely to remain a considerable budget requirement in the foreseeable future. While such efforts in providing compensation to affected families are invaluable due to the catastrophic financial burden snakebite inflicts,^5^ a more comprehensive state and/or national approach is required to significantly reduce snakebite-related deaths and disabilities, thus reducing the overall economic burden to the state. Appraisals of adequate snakebite care in West Africa demonstrates that improved access to snakebite care could have substantial economic benefits for countries in terms of the number of disability-adjusted life-years saved.^8,15^

While the interviews conducted with compensation recipients cannot be considered a robust qualitative study because of the limitations outlined in the Methods section, the profiles of the interviewees mirror the typical population demographics of those most at risk of snakebite: rural poverty, high levels of illiteracy and employment in agriculture or manual labour.^4,16^ When describing the incidents that led to the deaths of their relatives and their subsequent compensation claims, all of the participants described well-known snakebite risk factors immediately prior or post bite, such as sleeping on the floor, rodents being present in the house, working in agriculture, using traditional healers and other delays in accessing healthcare, all of which have been repeatedly demonstrated to result in an increased likelihood of mortality and morbidity after envenoming,^16–21^ and are likely to have been experienced by the majority of compensation claimants throughout the state.^22,23^ Investment in snakebite prevention and appropriate snakebite first aid education has been demonstrated to impact positively on incidences of snakebite and improved outcomes and will remain essential in reducing snakebite mortality.^24–26^ The interviewees revealed that compensation was often spent on home improvement, funeral expenses or debt repayment. While most interviewees were satisfied with the amount of compensation they received, two families had concerns about the long-term implications of the snakebite, such as reduced education provision for bereaved children, and the financial burden imposed, despite the compensation.

As mentioned above, obtaining reliable statistics of snakebite envenoming in India has been problematic.^27^ The absence of reliable metrics for evaluating snakebite incidence year on year makes assessing the impact of any intervention to reduce snakebite burden extremely difficult. The data presented here alongside previous reports demonstrating snakebite incidence are broadly consistent,^2^ indicating that the frequency of compensation payments due to snakebite death data may be a useful metric of the impact of any snakebite interventions designed to reduce mortality within Madhya Pradesh. There are three reasons for this: (i) claiming compensation for a snakebite death is not hospital dependent, and thus is capable of recording deaths outside hospital settings; (ii) to claim compensation the cause of death needs to be certified as due to snakebite via a postmortem report; and (iii), the well-documented financial burden of snakebite^5^ provides an incentive to report snakebite deaths. While the obvious disadvantage of this metric is only capturing the incidence of snakebite death, and not disability, and so will not be universal in its ability to capture bite incidences, any significant and sustained reduction in compensation payouts would be suggestive of effective interventions. Furthermore, compensation payout data can be interrogated to demonstrate areas within Madhya Pradesh that could be considered hotspots for snakebite (such as Sagar and Satna; Figure 1), and may assist in determining where snakebite-mitigation strategies should be prioritised or may assist in antivenom inventory management at a state level.

### Limitations and future research

There are limitations to the current study concerning the precision of the data provided, a factor which is outside the control of the authors, and the number of applications turned down for compensation disbursement. Absence of this information prevents comprehensive analysis of the compensation process, as the extent of application rejections and reasons cannot be assessed, and is an important area for further study. The data obtained from the Madhya Pradesh health authorities only detail the numbers of payments and the total amount paid for each district in a given period. Much more detailed information may be available that would allow further observation into other aspects of envenoming, such as seasonal trends and the age distribution of victims. In addition to the limitations regarding the interviews outlined in the Methods section, cultural and societal dynamics that prioritise male voices and perspectives over those of women may have influenced the accuracy of interviews conducted in families where women were the recipients of compensation. In such cases, the reported information may not fully represent the experiences of the women themselves.

### Conclusions

Using data of successful compensation payouts to families of deceased snakebite victims, we demonstrate a substantial and verified snakebite mortality incidence in Madhya Pradesh, India, resulting in large annual compensation payments from the state government. Interviews with a limited number of compensation recipients suggest that the events leading to their compensation claims were in part due to well-known risks and behaviours that contribute to poor outcomes in envenoming. The frequency of compensation payouts due to snakebite death could serve as a simple and reliable measure of the effectiveness of any snakebite-reduction strategies employed, such as improved community awareness and snakebite first aid schemes. This approach could be particularly effective if implemented alongside the National Action Plan for Snakebite Envenoming (NAP-SE) recently published by the Indian Government.^28^

## Supplementary Material

trae045_Supplemental_File

## Data Availability

The interview data underlying this article cannot be shared publicly due to the need to protect the privacy of individuals that participated in the study. All other data are available in the article and its accompanying supplementary material.
