# Corrigendum to: Reported snakebite mortality and state compensation payments in Madhya Pradesh, India, from 2020 to 2022

**DOI:** 10.1093/trstmh/trae099

**Published:** 2024-10-25

This is a corrigendum to: Priyanka Kadam, Bhupeshwari Patel, Maya Gopalakrishnan, Freston M Sirur, Omesh K Bharti, Amit Agrawal, Md Yunus, Dayal B Majumdar, Stuart Ainsworth, Reported snakebite mortality and state compensation payments in Madhya Pradesh, India, from 2020 to 2022, *Transactions of The Royal Society of Tropical Medicine and Hygiene*, 2024; trae045, https://doi.org/10.1093/trstmh/trae045

Since the originally published version of this paper, the author Freston M Sirur's affiliations have been corrected to clarify that they relate to ‘Kasturba Medical College, Manipal’.

