# Supplementary material for: Reported snakebite mortality and state compensation payments in Madhya Pradesh, India, from 2020 to 2022
Source: Trans R Soc Trop Med Hyg. 2024 Aug 6;119(2):158–65. doi: 10.1093/trstmh/trae045 (PMC11791426; doi:10.1093/trstmh/trae045)
Supplement: trae045_Supplemental_File [file trae045_supplemental_file.docx]

**File S1 Interview questionnaire and topic guide**

1. How was the person who died of snakebite related to you?
2. How old would they have been?
3. In which year did they die?
4. What was their occupation?
5. What is your occupation?
6. What is your monthly income?
7. How many earning people are there in your house?
8. How many members are there in the family?
9. How many children are there in the household?
10. Which tribal society do you belong to?
11. When and where did the snakebite occur?
12. Describe what happened immediately after the snakebite
13. Was traditional healing performed?
14. How long after the snakebite did the victim reach the health centre?
15. How did you take him to the hospital?
16. Which hospital was the victim admitted?
17. Was any medicine given at the hospital?
18. Did the victim show have any symptoms occur?
19. What time did the death occur?
20. What was the expense incurred for transportation and treatment?
21. Where did the post mortem take place?
22. How long did it take to receive the post mortem report?
23. When did you apply for compensation?
24. How long did the take to receive the compensation?
25. How much compensation was received?
26. Did you face any problems in applying or receiving compensation?
27. How was the compensation used?
28. Do you have any comments or thoughts on how the incident could have been prevented or what should be done to prevent a similar outcome for other victims?
